# Cysteine allows ovarian cancer cells to adapt to hypoxia and to escape from carboplatin cytotoxicity

**DOI:** 10.1038/s41598-018-27753-y

**Published:** 2018-06-22

**Authors:** Sofia C. Nunes, Cristiano Ramos, Filipa Lopes-Coelho, Catarina O. Sequeira, Fernanda Silva, Sofia Gouveia-Fernandes, Armanda Rodrigues, António Guimarães, Margarida Silveira, Sofia Abreu, Vítor E. Santo, Catarina Brito, Ana Félix, Sofia A. Pereira, Jacinta Serpa

**Affiliations:** 1 CEDOC, Chronic Diseases Research Centre, NOVA Medical School|Faculdade de Ciências Médicas, Universidade NOVA de Lisboa, Campo dos Mártires da Pátria, 130, 1169-056 Lisboa, Portugal; 2 Unidade de Investigação em Patobiologia Molecular do Instituto Português de Oncologia de Lisboa Francisco Gentil (IPOLFG), Rua Prof Lima Basto, 1099-023 Lisboa, Portugal; 3 Serviço de Oncologia Médica do Instituto Português de Oncologia de Lisboa Francisco Gentil (IPOLFG), Rua Prof Lima Basto, 1099-023 Lisbon, Portugal; 4 Serviço de Patologia Clinica do Instituto Português de Oncologia de Lisboa Francisco Gentil (IPOLFG), Rua Prof Lima Basto, 1099-023 Lisbon, Portugal; 5 Instituto de Biologia Experimental e Tecnológica, Apartado 12, 2780-901 Oeiras, Portugal; 6 Instituto de Tecnologia Química e Biológica António Xavier, Universidade Nova de Lisboa, Av. da República, 2780-157 Oeiras, Portugal; 7 Serviço de Anatomia Patológica do Instituto Português de Oncologia de Lisboa Francisco Gentil (IPOLFG), Rua Prof Lima Basto, 1099-023 Lisbon, Portugal

## Abstract

Ovarian cancer is the second most common gynaecologic malignancy and the main cause of death from gynaecologic cancer, due to late diagnosis and chemoresistance. Studies have reported the role of cysteine in cancer, by contributing for hydrogen sulphide (H_2_S) generation and as a precursor of glutathione (GSH). However, the role of cysteine in the adaptation to hypoxia and therapy response remains unclear. We used several ovarian cancer cell lines, ES2, OVCAR3, OVCAR8, A2780 and A2780cisR, to clarify cysteine relevance in ovarian cancer cells survival upon hypoxia and carboplatin. Results show that ES2 and OVCAR8 cells presented a stronger dependence on cysteine availability upon hypoxia and carboplatin exposure than OVCAR3 cells. Interestingly, the A2780 cisR, but not A2780 parental cells, benefits from cysteine upon carboplatin exposure, showing that cysteine is crucial for chemoresistance. Moreover, GSH degradation and subsequent cysteine recycling pathway is associated with ovarian cancer as seen in peripheral blood serum from patients. Higher levels of total free cysteine (Cys) and homocysteine (HCys) were found in ovarian cancer patients in comparison with benign tumours and lower levels of GSH were found in ovarian neoplasms patients in comparison with healthy individuals. Importantly, the total and S-Homocysteinylated levels distinguished blood donors from patients with neoplasms as well as patients with benign from patients with malignant tumours. The levels of S-cysteinylated proteins distinguish blood donors from patients with neoplasms and the free levels of Cys in serum distinguish blood from patients with benign tumours from patients with malignant tumours. Herein we disclosed that cysteine contributes for a worse disease prognosis, allowing faster adaptation to hypoxia and protecting cells from carboplatin. The measurement of serum cysteine levels can be an effective tool for early diagnosis, for outcome prediction and follow up of disease progression.

## Introduction

Ovarian cancer is a group of distinct diseases that have a common anatomical location^1^ and it is the major cause of death from gynaecologic cancer and the second most common gynaecologic malignancy worldwide^2,3^. The diagnosis at an advanced stage, when a cure is rare, together with resistance to conventional therapy, have a dramatic impact in patient survival^4^. Epithelial ovarian cancer (EOC) includes the majority of malignant ovarian neoplasms^5^, and the carcinoma histotypes are serous (OSC), endometrioid, clear cell (OCCC) and mucinous. The high-grade OSC is the prevalent histotype^4^ with diagnosis at an advanced stage in approximately 70% of patients^1^. The OCCC is a rather uncommon histotype that is frequently diagnosed at an initial stage but highly chemoresistant^6^. The standard care for ovarian cancer is a combination of surgery and paclitaxel-carboplatin combined chemotherapy^7^. However, despite an initial response, the disease recurs in over 85% of cases with advanced ovarian cancer^8^. The development of ascites is a common characteristic of ovarian cancer^9^. The ascitic fluid contains growth factors secreted by both cancer and stromal cells^9^ and these factors are mitogenic to cancer cells, contributing for an ideal microenvironment for tumour growth^10,11^.

Metabolism reprogramming is a common feature of cancer cells, providing enough sources of energy and biomass to support cancer cell survival and proliferation^12^. Serpa and Dias proposed a model in which cells not adapted to microenvironment would undergo cell death whereas cells metabolically fitted would be positively selected and carry on cancer progression and metastasis^13^. Soon after this report, Hanahan and Weinberg also included reprogramming of energy metabolism as an emerging hallmark of cancer^14^.

Expanding evidence exists on the dependence of these processes on cysteine and its metabolism, as cysteine contributes to the generation of hydrogen sulphide (H_2_S)^15–20^ and glutathione (GSH)^21–23^. It is known that tumours are subjected to intermittent hypoxia^24,25^ and that hypoxia-inducible factors (HIFs) mediate adaptive pathophysiological responses underlying resistance to radiation therapy and chemotherapy^26^. In the context of ovarian cancer, Cutter *et al*.^27^, have recently reported that cancer cell lines subject to hypoxia are more invasive, have increased migratory ability and an epithelial-mesenchymal transition (EMT) phenotype^27^. Additionally, higher levels of cytoplasmic thiol-containing species, including GSH or metallothioneins have been associated with resistance to platinum-based chemotherapy^23,28^. These species are rich in cysteine and result in decreased drug availability and increased intracellular drug detoxification/inactivation, since platinum binds readily to sulfhydryl groups^29^. Lopes-Coelho *et al*.^23^ have shown that different ovarian cancer histological types present different metabolic outcomes concerning thiols and chemoresistance. Under normoxic conditions the OCCC cells were more resistant to carboplatin than OSC cells. Moreover, the inhibition of GSH synthesis by buthionine sulphoximine (BSO) sensitized OCCC cells to carboplatin^23^. Therefore, chemoresistance in those cells might be associated with thiols dynamics. However, the role of cysteine in hypoxic ovarian cancer cells survival and its influence on cells’ response to chemotherapy remains unknown. As hypoxia can be a mean of selecting more aggressive resistant cancer cells, we hypothesised that cysteine favours the adaptation to hypoxic conditions and chemotherapeutic agents, having a pivotal role in tumour progression, recurrence and chemoresistance. To test this hypothesis, we used several ovarian cancer cell lines, including serous carcinoma (OSC - OVCAR3 and OVCAR8), sensitive and resistant to Cisplatin A2780 (A2780 parental and A2780 cisR) unspecified histotype ovarian carcinoma cells and clear cell carcinoma (OCCC-ES2) cells. To better clarify the relevance of cysteine and other thiols in ovarian cancer, we analysed their levels in peripheral blood serum from patients with benign and malignant tumours, from healthy individuals and in ascitic fluid from ovarian cancer patients.

## Results

### Cysteine has a widespread protective effect under hypoxia in ovarian cancer cells

**Figure 1:**
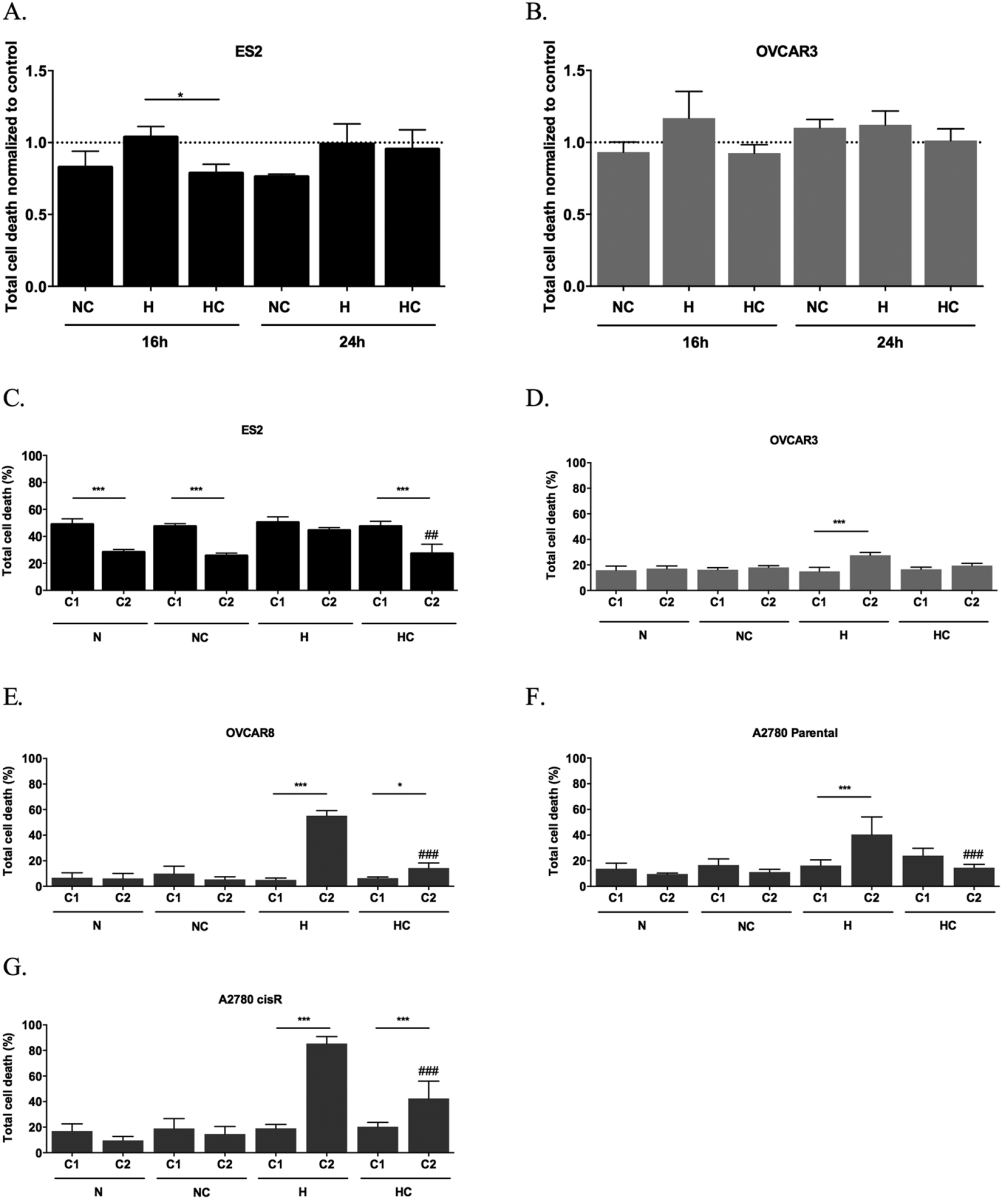
Cysteine has a widespread protective effect under hypoxia in ovarian cancer cells. Total cell death normalized by control for 16 and 24 h of assay for (**A**) ES2 cells and (**B**) OVCAR3 cells and total cell death in the first cycle (C1) and in the second cycle (C2) of treatments in (**C**) ES2, (**D**) OVCAR3, (**E**) OVCAR8, (**F**) A2780 parental cells and (**G**) A2780 cisR cells. N - Normoxia; NC - Normoxia supplemented with cysteine; H - Hypoxia; HC - Hypoxia supplemented with cysteine. Results are shown as mean ± SD. Cardinals represent statistical significance among hypoxia with and without cysteine supplementation within the same cycle of treatments. *p < 0.05, **p < 0.01, ***p < 0.001 (One-way ANOVA with post hoc Tukey tests).

Cysteine protected ES2 cells from death under hypoxia (H) after 16 h (*p* = 0.026) (Fig. 1A), but the effect was not maintained after 24 h (Fig. 1A). Regarding OVCAR3 cells, no differences were found in total cell death levels in both time-points (Fig. 1B). However, in early apoptosis (*p* = 0.01) after 24 h of assay cysteine was advantageous for cells under H (Suppl. Fig. 1).

The effect of normoxia (N) with cysteine and H without cysteine in total cell death was similar between cell lines. However, there was no similarity for the effect of H plus cysteine on cell death in ES2 and OVCAR3 cells (*p* = 0.049). As observed, ES2 cells presented lower death levels compared to its control than OVCAR3 cells (Fig. 1A and B). With 24 h of incubation under N, cysteine induced higher levels of cell death in OVCAR3 cells compared to ES2 cells (*p* = 0.001). However under H, there were no significant differences among cell lines.

Next, we addressed the long-term effect of cysteine in ovarian cancer cells, also clarifying if the absence of protection by cysteine under H with 24 h of assay in ES2 was due to cysteine consumption or CoCl_2_ exhaustion. The exposure of ES2 cells to two experimental cycles revealed a significant decrease in total cell death levels in all conditions from the first to the second cycle (*p* = 0.000 for N, NC and HC), with the exception of H without cysteine (Fig. 1C).

No major alterations were registered from the first to the second cycle in total cell death for OVCAR3 cell line with the exception of total cell death under H without cysteine (*p* < 0.001; Fig. 1D).

We also investigated the effect of cysteine in the adaptation to hypoxia in other ovarian cancer cell lines. In OVCAR8 cell line, total cell death levels increased from the first to the second cycle under hypoxia, especially without cysteine, with no differences in the normoxia conditions (under H *p* < 0.001, under HC *p* = 0.020) (Fig. 1E). Nonetheless, in the second, cysteine revealed to be also advantageous for this cell line (H vs HC *p* < 0.001).

We also addressed if cysteine impacts differently the adaptive potential to hypoxia in sensitive and cisplatin resistant A2780 cells. In hypoxia condition, both A2780 and A2780 cisR cell lines died more at the second cycle of culture without cysteine and A2780 cisR with cysteine (A2780 cells *p* < 0.001; A2780 cisR cells *p* < 0.001 under H and HC). Nevertheless, cysteine revealed to be advantageous under hypoxia in the second cycle for both cell lines (H vs HC for A2780 parental cells *p* < 0.001, for A2780 cisR cells *p* < 0.001) (Fig. 1F and G).

So different ovarian cancer cells present different abilities to deal with metabolize cysteine Moreover, results have shown a widespread protective effect of cysteine upon hypoxic stress in ovarian cancer cells from different origins.

### The protective effect of cysteine under hypoxia (H) in ES2 (OCCC) is dose dependent

**Figure 2:**
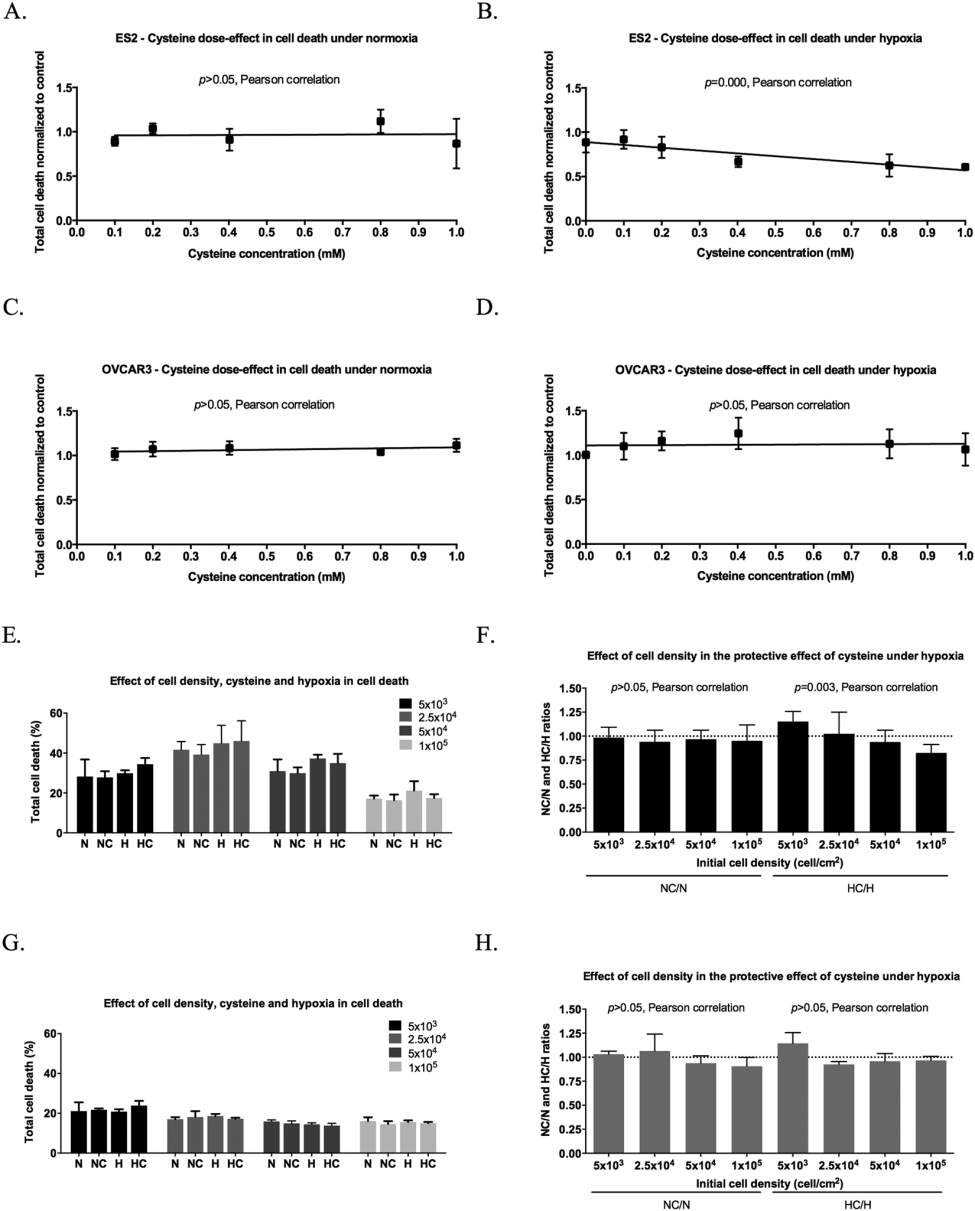
The dose dependent and cell density dependent effects of cysteine in hypoxia adaptation in ES2 (OCCC) and OVCAR3 (OSC) cells. (**A**) Pearson correlation under normoxia for ES2 cell line. (**B**) Pearson correlation under hypoxia for ES2 cell line. (**C**) Pearson correlation under normoxia for OVCAR3 cell line and (**D**) Pearson correlation under hypoxia for OVCAR3 cell line for 16 h of assay. Treatments were normalized by the control values (normoxia without cysteine supplementation). (**E**) Percentage of total cell death for ES2 cells for 16 h of assay for different initial cell densities. (**F**) Pearson correlation for NC/N and HC/H ratios for ES2 cells. (**G**) Percentage of total cell death for OVCAR3 cells for 16 h of assay for different initial cell densities and (**H**) Pearson correlation for NC/N and HC/H ratios for OVCAR3 cells. N – Normoxia; NC – Normoxia supplemented with cysteine; H – Hypoxia; HC – Hypoxia supplemented with cysteine. 5 × 10^3^, 2.5 × 10^4^, 5 × 10^4^ and 1 × 10^5^ refers to initial cell densities expressed as cells/cm^2^. Results are shown as mean ± SD.

To test whether the protective effect of cysteine in ES2 cells in H was dose-dependent, we performed an assay under N and H without cysteine and with increasing cysteine concentrations. While there were no dose-dependence effects in ES2 in N (Fig. 2A), cell death decreased in H with increasing cysteine concentrations (Pearson r = −0.777, *p* < 0.001) (Fig. 2B).

Regarding OVCAR3 cells, there was no correlation between cell death and cysteine concentration neither under N nor under H (Fig. 2C and D).

### The protective effect of cysteine under hypoxia (H) in ES2 (OCCC) is cell density-dependent

We also asked if this protective effect of cysteine under H in ES2 cells was dependent on cell density. In a general way, results showed a decreased cell death trend with increased cell density (*p* < 0.001 for all conditions) (Fig. 2E). Moreover, results suggest that the protective effect of cysteine under H is dependent on cell density, as demonstrated by the decreased cell death ratio HC/H with the increase of cell density (Pearson r = −0.628, *p* = 0.003) (Fig. 2F) whereas cell density did not impact on the cell death ratio NC/N (Fig. 2F).

Regarding OVCAR3 cells, the results also showed lower levels of cell death with higher cell densities in all conditions (*p* = 0.004), with the exception of the control (Fig. 2G). However, there was no impact of cell density neither on the NC/N nor on the HC/H ratios (Fig. 2H).

### Cysteine increases mitochondrial membrane potential in ES2 (OCCC) under normoxia and in OVCAR3 (OSC) under normoxia and hypoxia

Mitochondrial membrane potential (Δψm) is associated with ATP production by oxidative phosphorylation, being a good indicator of cells health and functional status^30^. Δψm changes can be measured by red/green fluorescence intensity ratio using JC-1 probe, in which Δψm loss can be detected by a decrease in this ratio.

**Figure 3:**
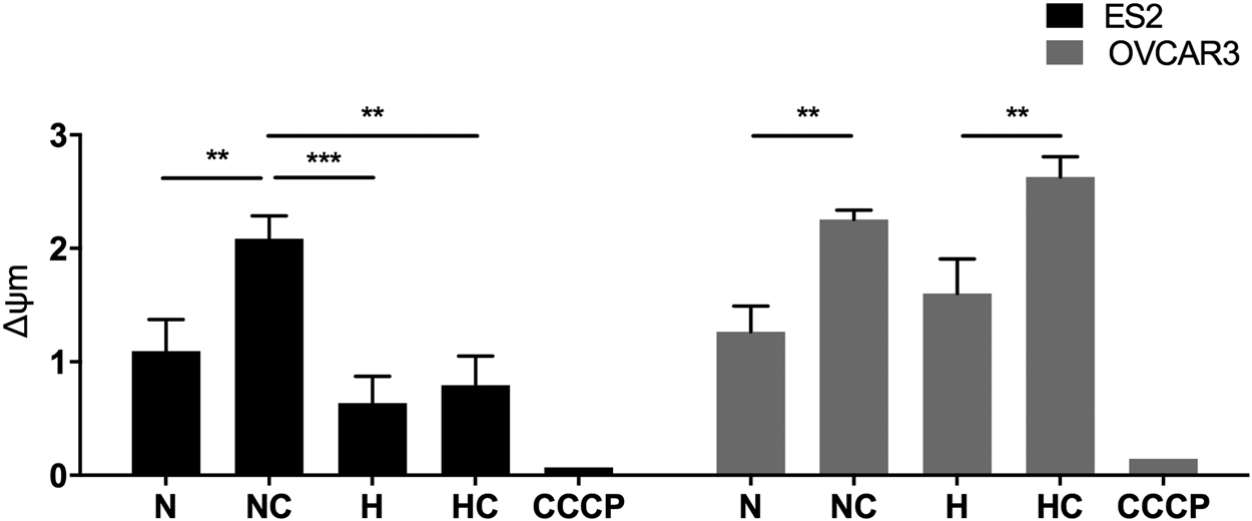
Mitochondrial membrane potential under normoxia and hypoxia with and without cysteine, in ES2 (OCCC) and OVCAR3 (OSC) cells. Quantification of Δψm expressed as a ratio of red/green fluorescence intensity ratio in the different treatments (ratio of geometrical means) for ES2 and OVCAR3 cell lines for 16 h of assay. CCCP is a positive control for depolarization. N – Normoxia; NC – Normoxia supplemented with cysteine; H – Hypoxia; HC – Hypoxia supplemented with cysteine. Results are shown as mean ± SD. *p < 0.05, **p < 0.01, ***p < 0.001 (One-way ANOVA with post hoc Tukey tests).

For ES2 cells, the Δψm loss was lower under N plus cysteine compared to the other conditions (*p* = 0.005 compared to N, *p* < 0.001 compared to H and *p* = 0.001 compared to HC) (Fig. 3). However, there were no differences among H with and without cysteine.

Regarding OVCAR3 cells, the Δψm loss was always lower in the presence of cysteine (NC vs N *p* = 0.002, NC vs H *p* = 0.024, HC vs N *p* < 0.001 and HC vs H *p* = 0.002) (Fig. 3).

Together, results suggest that cysteine allows mitochondrial function and cell health, especially under normoxia for ES2 cells and both under normoxia and hypoxia for OVCAR3 cells.

### ES2 (OCCC) adaptation to hypoxia relies on free intracellular cysteine availability

Cysteine intracellular pool has the contribution of the free cysteine fraction and the protein-bound fraction (S-cysteinylated proteins-CysSSP).

**Figure 4:**
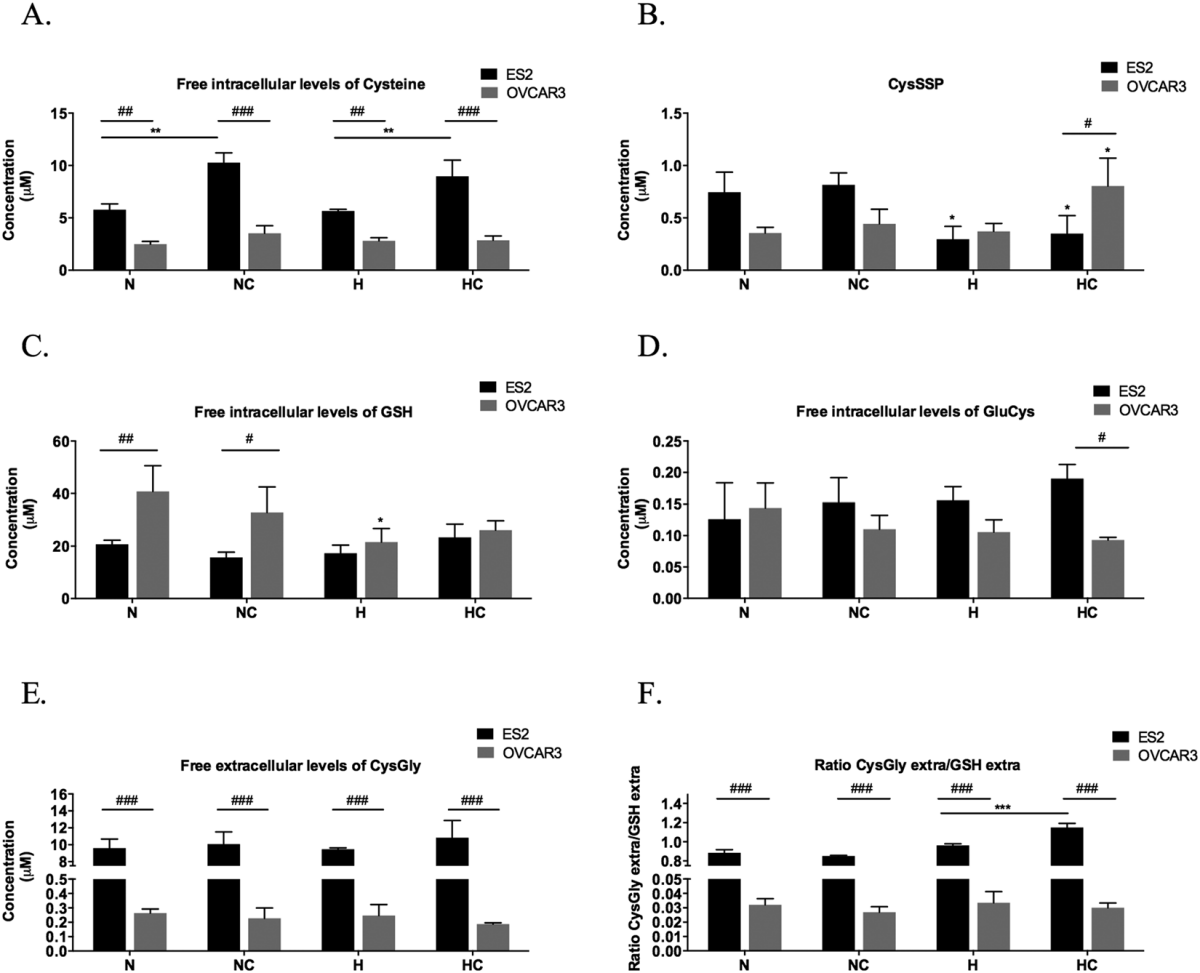
ES2 (OCCC) adaptation to hypoxia relies on free intracellular cysteine availability. Free intracellular levels of (**A**) Cys– cysteine, (**B**) S-cysteinylated proteins – CysSSP, (**C**) GSH – Glutathione, (**D**) GluCys – Glutamylcystein, (**E**) CysGly – Cysteinylglycine and (**F**) free extracellular CysGly/free extracellular GSH ratio in ES2 (black bars) and OVCAR3 (grey bars) cells. N – Normoxia; NC – Normoxia supplemented with cysteine; H – Hypoxia; HC – Hypoxia supplemented with cysteine. Results are shown as mean ± SD. Cardinals represent statistical significance between cell lines. Asteriscs represent statistical significance among treatments within the same cell line or in comparison with the control (normoxia without cysteine supplementation). *p < 0.05, **p < 0.01, ***p < 0.001 (One-way ANOVA with post hoc Tukey tests).

ES2 (OCCC) cells presented higher free intracellular levels of cysteine in all treatments than OVCAR3 (OSC) cells (*p* = 0.001 in N, *p* < 0.001 in NC, *p* = 0.003 in H, *p* < 0.001 in HC) (Fig. 4A). Also, cysteine supplementation induced higher free intracellular levels of cysteine both in N (*p* = 0.001) and in H (*p* = 0.008). This result might indicate that H did not impair ES2 cells capacity to uptake this amino acid or that in this cells cysteine is not channelled for protein-S-cysteinylation pool or to *de novo* GSH synthesis.

Thus, we asked how CysSSP was comparable between the two cell lines. We observed that hypoxia reduces CysSSP in ES2 cells (*p* = 0.02, for N vs H, *p* = 0.037 for N vs HC, *p* = 0.009 for NC vs H and *p* = 0.016 for NC vs HC) (Fig. 4B). This might suggest that this intracellular pool of cysteine is reduced to supply free cysteine for cells adaptation to hypoxia. In fact, cysteine in these cells does not increase CysSSP indicating the need of free cysteine availability and that the supplemented cysteine is not targeting proteins by S-cysteinylation.

To clarify if this will be important for maintaining GSH pool, we next evaluated the effect of tested conditions in GSH availability. No changes were observed for GSH intracellular levels in conditions tested (Fig. 4C). Results suggest that ES2 maintain high levels of available cysteine that are not being canalized to GSH synthesis as no changes were observed for GluCys, the first product of GSH synthesis, even with supplementation of cysteine (Fig. 4D). Moreover, in hypoxia conditions, the supplementation of cysteine increased GSH catabolism and cysteine recycling, as seen by the increased ratio CysGly/GSH, being CysGly the first product of GSH degradation. Thus, hypoxia decreases CysSSP supplying free Cys (Fig. 4B) and cysteine supplementation in hypoxia increases intracellular pool of free Cys (Fig. 4A) and the recycling of cysteine through GSH catabolism (Fig. 4F). In accordance, the protective effect of cysteine under hypoxia was dependent on its concentration and cell metabolic profile, indicating that intracellular pool of free cysteine in ES2 plays an important role in adaptation to hypoxia.

A different scenario was observed in OVCAR3. This cell line presented lower intracellular levels of cysteine than ES2 and the supplementation of cysteine did not have any effect on its free intracellular levels (Fig. 4A). This might suggest alteration in cysteine import or that imported cysteine is being channelled for CySSP pool or GSH synthesis. In addition, in this cell line H did not changed CysSSP and cells exposed to cysteine supplementation and H experienced an increase in CysSSP (*p* = 0.032 compared to N and *p* = 0.038 compared to H) (Fig. 4B). Also, under H with cysteine, ES2 cells presented lower levels of S-cysteinylated proteins (CysSSP) than OVCAR3 cells (*p* = 0.030) (Fig. 4B). Moreover, the recycling of cysteine through GSH catabolism is much lower than in ES2 as evaluated by the ratio CysGly/GSH (*p* < 0.001) (Fig. 4F). Together, results suggest that OVCAR3 are not so dependent on free cysteine as ES2.

### Cysteine and hypoxia (H) have a role on the response to carboplatin by ovarian cancer cells

Usually, ovarian cancer therapy protocols include platinum salts and taxanes^7^. However, we tested paclitaxel with and without carboplatin and no relevant alterations were found related to H (Suppl. Fig. 2). In N it was only observed an increase in cell death due to paclitaxel in the presence of cysteine in the first cycle of ES2 cells exposure to drugs (Suppl. Fig. 2). So, the experimental course was developed using carboplatin, as chemically thiols react directly with platinum salts (oxidative compounds) and under H a protective effect was noticed in OCCC cells which are intrinsically resistant to carboplatin^23^.

Herein, we addressed the role of H and cysteine in ovarian cancer cells response and adaptation to carboplatin, by exposing cells to two cycles of carboplatin and assessing the effects on cell death from the first to the second cycle of drug exposure.

**Figure 5:**
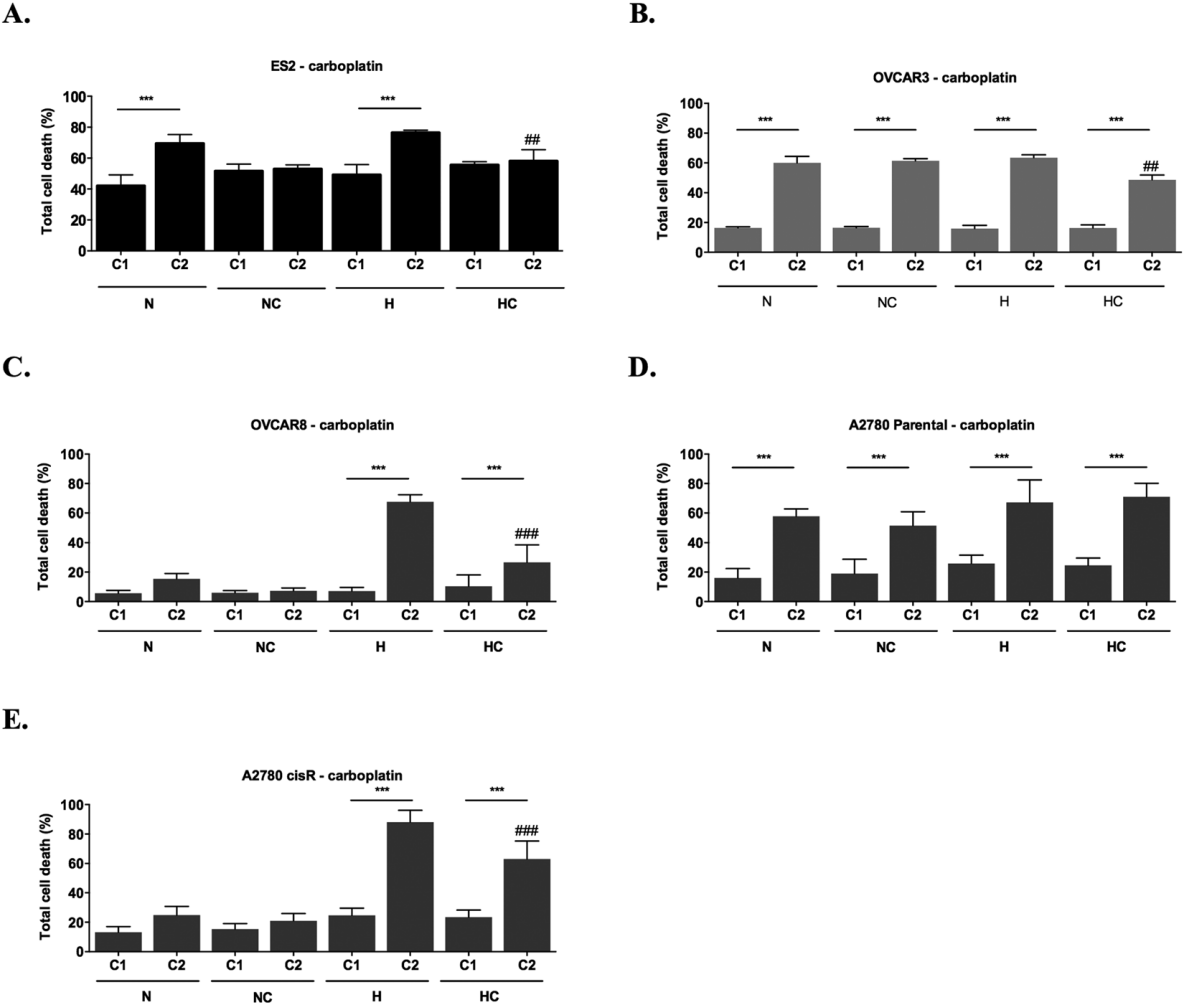
Cysteine and hypoxia (H) have a role on the response to carboplatin by ovarian cancer cells. Total cell death in the first (C1) and in the second (C2) cycles of treatments in the presence of Carboplatin for (**A**) ES2, (**B**) OVCAR3, (**C**) OVCAR8, (**D**) A2780 parental cells and (**E**) A2780 cisR cells. N – Normoxia; NC – Normoxia supplemented with cysteine; H – Hypoxia; HC – Hypoxia supplemented with cysteine. Results are shown as mean ± SD. Cardinals represent statistical significance among hypoxia with and without cysteine supplementation within the same cycle of treatments. *p < 0.05, **p < 0.01, ***p < 0.001 (One-way ANOVA with post hoc Tukey tests).

With one cycle of carboplatin, no differences were found among treatments in ES2 cells (Fig. 5A). Regarding the effect of two cycles of carboplatin, it was possible to observe differences among treatments (*p* = 0.002). Results showed that cysteine protects cells from carboplatin-induced death both in H and N (*p* = 0.008 in H and *p* = 0.015 in N) (Fig. 5A). When analysing the alterations in ES2 total cell death in consecutive cycles, results showed that carboplatin induced higher cell death at the second cycle only in the absence of cysteine treatment (*p* < 0.001 for N and H) (Fig. 5A). This result suggests that for ES2 cells, cysteine is not only a protective factor from death under H but it also allows faster adaptation to carboplatin both in N and H.

In OVCAR3 cells (Fig. 5B), with one cycle of carboplatin there were no differences among treatments in cell death levels. However, with two cycles, there were differences among conditions (*p* = 0.001), in which cell death levels were lower under H supplemented with cysteine compared to the other treatments (*p* = 0.007 compared to N; *p* = 0.003 compared to NC and *p* = 0.001 compared to H). Regarding changes from the first to the second cycle of carboplatin in OVCAR3 cells, there was an increase in total cell death in all treatments in the second cycle (*p* < 0.001) (Fig. 5B). In this cell line, under hypoxia, cysteine protected cells from death in the second cycle of carboplatin (*p* = 0.001). Nevertheless, total cell death was higher in this cycle compared to the first one (*p* < 0.001). Under N, cysteine did not confer any advantage for cells in the presence of carboplatin in the second cycle.

We also asked the effect of hypoxia and cysteine in other ovarian cancer cell lines response to carboplatin. For OVCAR8 cells, no differences were observed with one cycle of carboplatin exposure among experimental conditions (Fig. 5C). However, with two cycles, hypoxia was especially disadvantageous without cysteine for OVCAR8 cells (N vs H *p* < 0.001, NC vs H *p* < 0.001, N vs HC *p* = 0.041, H vs HC *p* < 0.001). When analysing the alterations in total cell death levels from the first to the second cycle in OVCAR8 cells, increased levels were observed under hypoxia with and without cysteine (under H and HC *p* < 0.001), without significant differences under normoxia (Fig. 5C). Nevertheless, cysteine was advantageous under hypoxia in the second cycle (H vs HC *p* < 0.001).

For A2780 parental cells, in the first cycle, no differences were observed among treatments in the presence of carboplatin. In the second cycle of experimental conditions, increased cell death levels were observed under hypoxia with cysteine (*p* = 0.02) compared to normoxia with cysteine. When analysing the alterations in total cell death levels from the first to the second cycle, results showed an increase in cell death between cycles in all the treatments (Fig. 5D).

Regarding A2780 cisR cells, in the first cycle it was observed an increase in cell death levels under hypoxia with and without cysteine supplementation compared to the control and to normoxia with cysteine (N vs H *p* = 0.001, N vs HC *p* = 0.005, NC vs H *p* = 0.007 and NC vs HC *p* = 0.026), with no differences in the other conditions (Fig. 5E). In the second cycle, hypoxia was disadvantageous for these cells, especially without cysteine (N/NC vs H/HC, *p* < 0.001). When analysing the alterations in total cell death levels from the first to the second cycle, results showed increased cell death levels only under hypoxia, both with (*p* < 0.001) and without (*p* < 0.001) cysteine (Fig. 5F). Nevertheless, cysteine was advantageous in the second cycle of experimental conditions for A2780 cisR under hypoxia also in the presence of carboplatin (H vs HC, *p* < 0.001), in contrast to A2780, the parental cell line.

Altogether, results revealed a positive effect of cysteine under hypoxia and in the presence of carboplatin in ovarian cancer cells from different origins, thus suggesting a widespread role for cysteine in the selection of more aggressive phenotypes in ovarian cancer cells.

### In ES2, OVCAR8 and A2780 cancer stem CD133^+^ cells command resistance to hypoxia and carboplatin

We next asked if the cancer stem CD133^+^ cells could be responsible for cell resistance to carboplatin.

For that, we compared the changes from the first cycle to the second cycle of treatments in the levels of CD133^+^ cells, based on the intensity of fluorescence.

**Figure 6:**
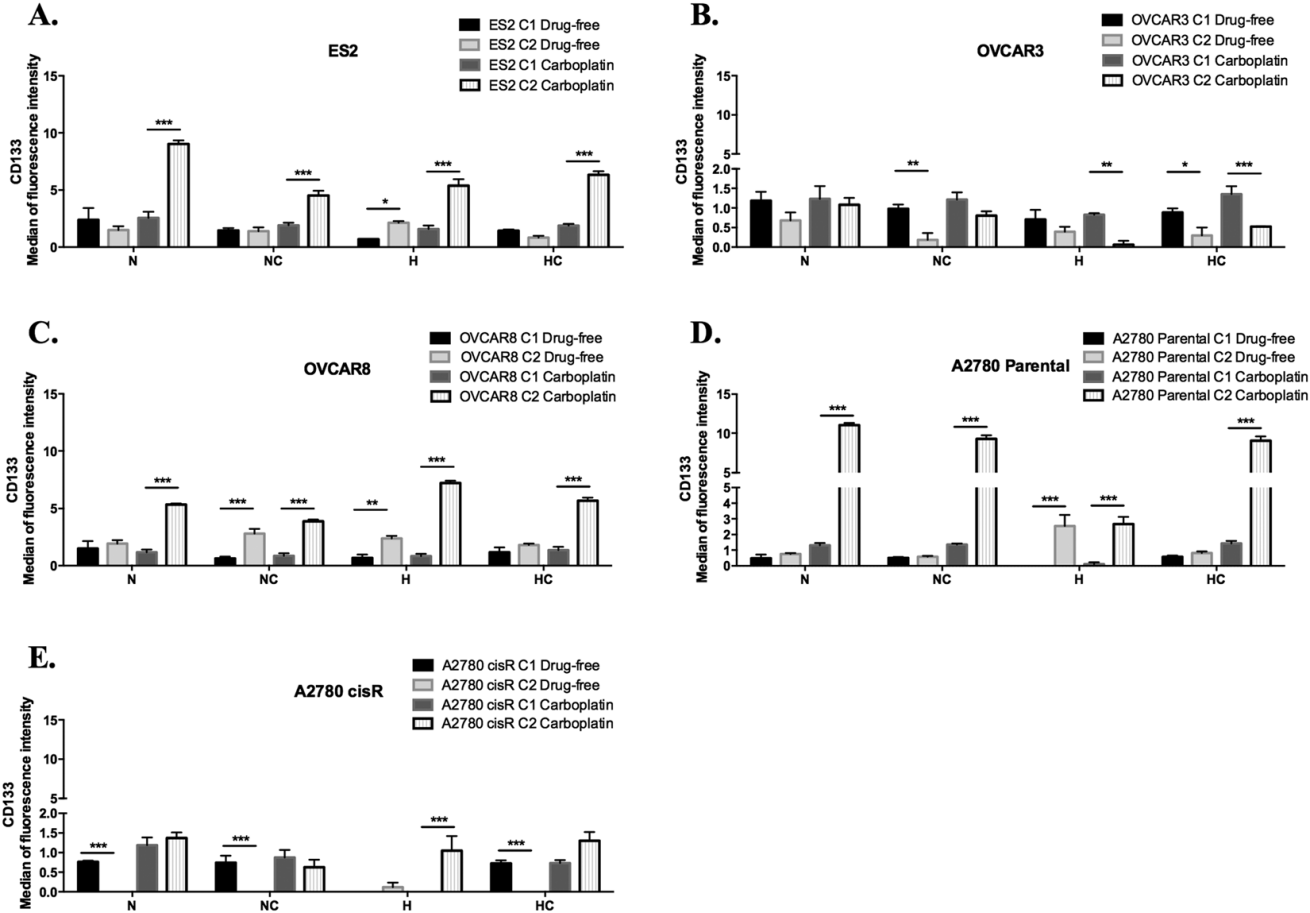
In ES2, OVCAR8 and A2780 cancer stem CD133^+^ cells command resistance to hypoxia and carboplatin. CD133 expression levels in a drug-free environment and upon carboplatin exposure for (**A**) ES2 cells, (**B**) OVCAR3 cells, (**C**) OVCAR8 cells, (**D**) A2780 parental cells and (**E**) A2780 cisR cells in the first and in the second cycles of treatments. N – Normoxia; NC – Normoxia supplemented with cysteine; H – Hypoxia; HC – Hypoxia supplemented with cysteine. Results are shown as mean ± SD. Asterisks represent statistical significance among the first and the second cycle within the same treatment. *p < 0.05, **p < 0.01, ***p < 0.001 (One-way ANOVA with post hoc Tukey tests).

In ES2 in a drug-free environment, results showed that hypoxia induced CD133 expression (Fig. 6) (p = 0.013). Upon carboplatin exposure, from the first cycle to the second cycle, there was an increase in CD133 expression in all treatments (under N, NC, H and HC *p* < 0.001) (Fig. 6A).

Interestingly, for OVCAR3 cells and in a drug-free environment, CD133 levels decreased in treatments with cysteine (under NC *p* = 0.001; under HC *p* = 0.02). Upon carboplatin exposure, there was a decrease in CD133 expression levels in treatments under hypoxia (under H *p* = 0.001; under HC *p* < 0.001), with no differences among cycles in treatments under normoxic conditions (Fig. 6B).

Regarding OVCAR8 cells, in a drug-free environment, results showed that hypoxia without cysteine induced CD133 expression (*p* = 0.001). Moreover, this effect was also observed under normoxia with cysteine supplementation (*p* < 0.001) (Fig. 6C). Upon carboplatin exposure, from the first cycle to the second cycle, there was an increase in CD133 expression in all treatments (under N, NC, H and HC *p* < 0.001) (Fig. 6C).

A2780 parental cells, in a drug-free environment, express more CD133 in H without cysteine (*p* < 0.000), with no significant differences among cycles in the other conditions (Fig. 6D). Upon carboplatin exposure, from the first cycle to the second cycle, an increase in CD133 expression was observed in all conditions (under N, NC, H and HC *p* < 0.001) (Fig. 6D). A different scenario was observed for A2780 cisR cells. In a drug-free environment, there was a decrease of CD133 expression from the first to the second cycle in all conditions (under N, NC and HC *p* < 0.001) (Fig. 6E) with the exception of hypoxia, in which there were no significant differences among cycles (Fig. 6E). Upon carboplatin exposure, hypoxia without cysteine induced CD133 levels (*p* < 0.001) from the first to the second cycle. Under hypoxia with cysteine, there was a trend to increase CD133 expression among cycles (*p* = 0.053) (Fig. 6E).

Together, results show that the levels of CD133 do not influence OVCAR3 cells resistance to neither hypoxia nor carboplatin, contrarily to what was observed in ES2, OVCAR8 and A2780 parental cells. Regarding the already resistant A2780 cisR cells, results point out that overall carboplatin resistance is not related with CD133 levels, only when combined with hypoxic stress.

### All thiols but GSH are increased in patients with ovarian tumours- high GSH turnover

**Figure 7:**
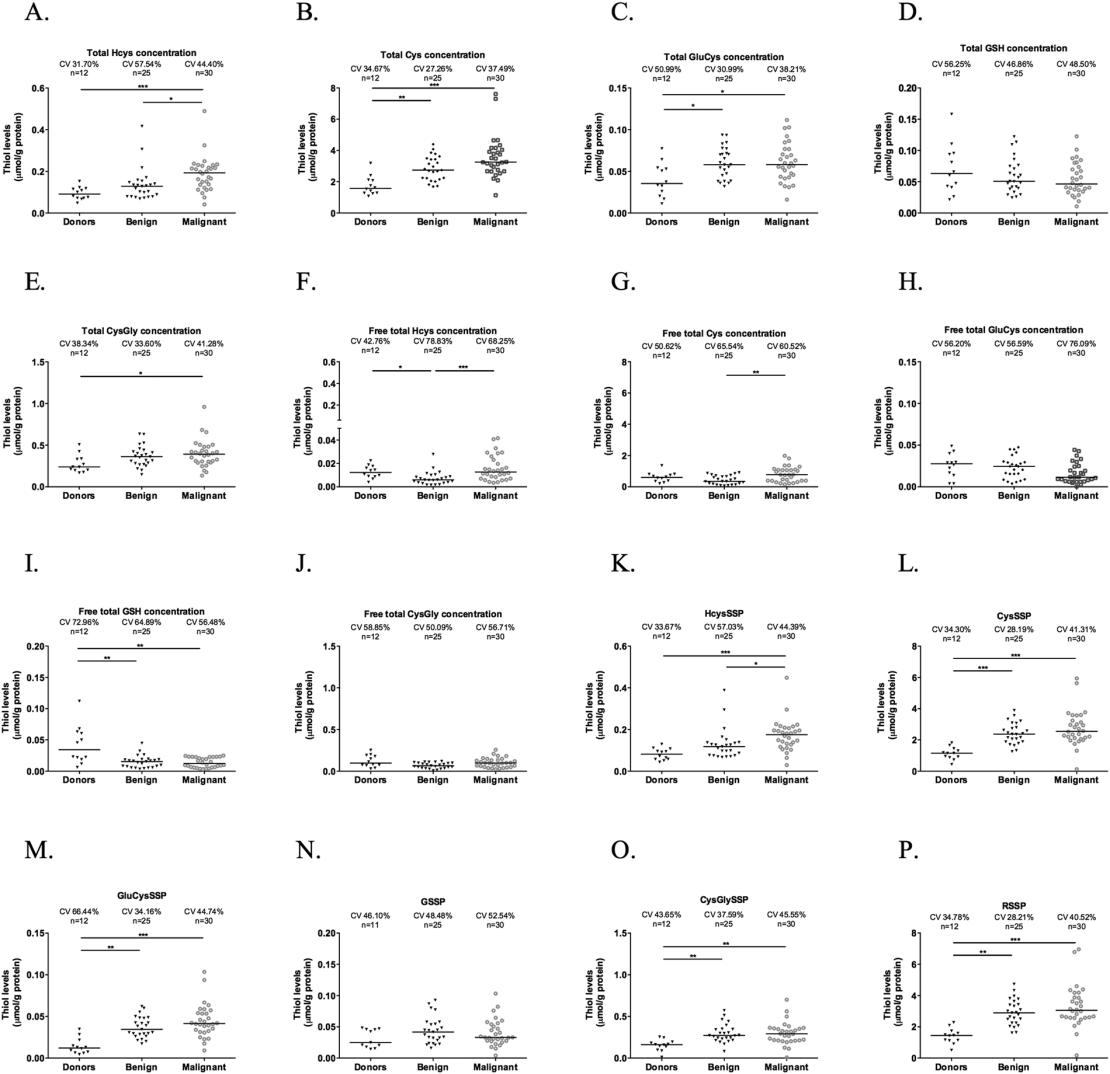
All thiols but GSH are increased in patients with ovarian tumours- high GSH turnover. Total thiols quantification in serum from healthy individuals (donors) and in serum from patients with benign and malignant ovarian tumours for (**A**) HCys- Homocysteine, (**B**) Cys – cysteine, (**C**) GluCys – Glutamylcysteine, (**D**) GSH – Glutathione, (**E**) CysGly – Cysteinylglycine. Thiol concentration was normalized by protein concentration. Free total thiols quantification in serum from healthy individuals (donors) and in serum from patients with benign and malignant ovarian tumours for (**F**) HCys- Homocysteine, (**G**) Cys – cysteine, (**H**) GluCys – Glutamylcysteine, (**I**) GSH – Glutathione, (**J**) CysGly – Cysteinylglycine. Thiol concentration was normalized by protein concentration. Thiols concentration bound to proteins quantification in serum from healthy individuals (donors) and in serum from patients with benign and malignant ovarian tumours for (**K**) HcySSP *– S*-*homocysteinylated* proteins, (**L**) CysSSP *– S*-*cysteinylated* proteins, (**M**) GluCysSSPS – *S*-*Glutamylcysteinylated* proteins, (**N**) GSSP *– S*-*glutathionylated* proteins, (**O**) CysGlySSP – *S*-*cysteinylglycinylated* proteins. (**P**) RSSP – total *S*-*thiolated* proteins. Thiol concentration was normalized by protein concentration. Results are shown as median. The *represent the statistical significance among groups. *p < 0.05, **p < 0.01, ***p < 0.001 (independent samples Kruskal Wallis One-way ANOVA with multiple comparisons).

Given the role of cysteine in the adaptation of ovarian cancer cell lines to hypoxic conditions and carboplatin exposure, we next assessed if higher serum levels of thiols were associated with ovarian tumours malignancy. Strikingly, total HCys levels were effective in distinguishing patients with malignant tumours from benign tumours and both of them from healthy individuals (Fig. 7A). Results showed that increased total levels of Cys and GluCys (GSH precursor) were associated with ovarian tumours regardless malignancy (Fig. 7B and C). Total GSH levels did not differ among groups (*p* > 0.05) and CysGly (GSH degradation product) was increased in patients with malignant tumours compared only to healthy individuals (*p* = 0.028) (Fig. 7D and E). Regarding free thiol content in plasma, Cys levels were increased in patients with malignant tumours compared to patients with benign tumours (*p* = 0.005) but without significant differences compared to healthy individuals (Fig. 7G).

Free HCys levels were able to distinguish patients with malignant tumours from benign tumours (*p* < 0.001) and patients with benign tumours from healthy individuals (*p* = 0.02) without significant differences between malignant tumours and healthy individuals (Fig. 7F). Lower free GSH levels were associated with ovarian neoplasms regardless malignancy (*p* = 0.006 donors vs benign tumours and *p* = 0.005 donors vs malignant tumours) (Fig. 7I). Free GluCys and free CysGly levels did not differ among groups (*p* > 0.05) (Fig. 7H and J).

Higher levels of S-cysteinylated, S-glutamylcysteinylated, and S-cysteinylglycinylated proteins (CysSSP, GluCysSSP and CysGlySSP) were associated with ovarian neoplasms regardless malignancy (CysSSP: donors vs benign *p* < 0.001, donors vs malignant *p* < 0.001; GluCysSSP: donors vs benign *p* = 0.001, donors vs malignant *p* < 0.001; CysGlySSP: donors vs benign *p* = 0.001, donors vs malignant *p* = 0.001) (Fig. 7L,M and O). Higher levels of S-Homocysteinylated proteins (HCysSSP) were associated only with malignant ovarian tumours (*p* = 0.019 benign vs malignant, *p* < 0.001 donors vs malignant) (Fig. 7K). There were no differences in levels of S-glutathionylated proteins (GSSP) among groups (Fig. 7N).

With the exception of GSH total levels, in which there was a trend to lower levels in serum from patients with ovarian neoplasms compared to healthy individuals, other thiols were mainly in the protein bound form and not free in plasma. Together, results suggested that higher levels of thiols are associated with ovarian neoplasms, regardless malignancy, showing that patients with ovarian neoplasms present different thiol dynamics.

Noticeably, total CysSSP was capable of distinguishing serum of healthy donors from patients with ovarian tumours (benign and malignant) and total free Cys levels distinguished serum of patients with malignant tumours from patients with benign tumours. Cysteine, free and protein- bound, are for sure a suitable marker for ovarian cancer.

### Cysteine is the prevalent thiol in ascitic fluid from patients with advanced ovarian cancer

**Figure 8:**
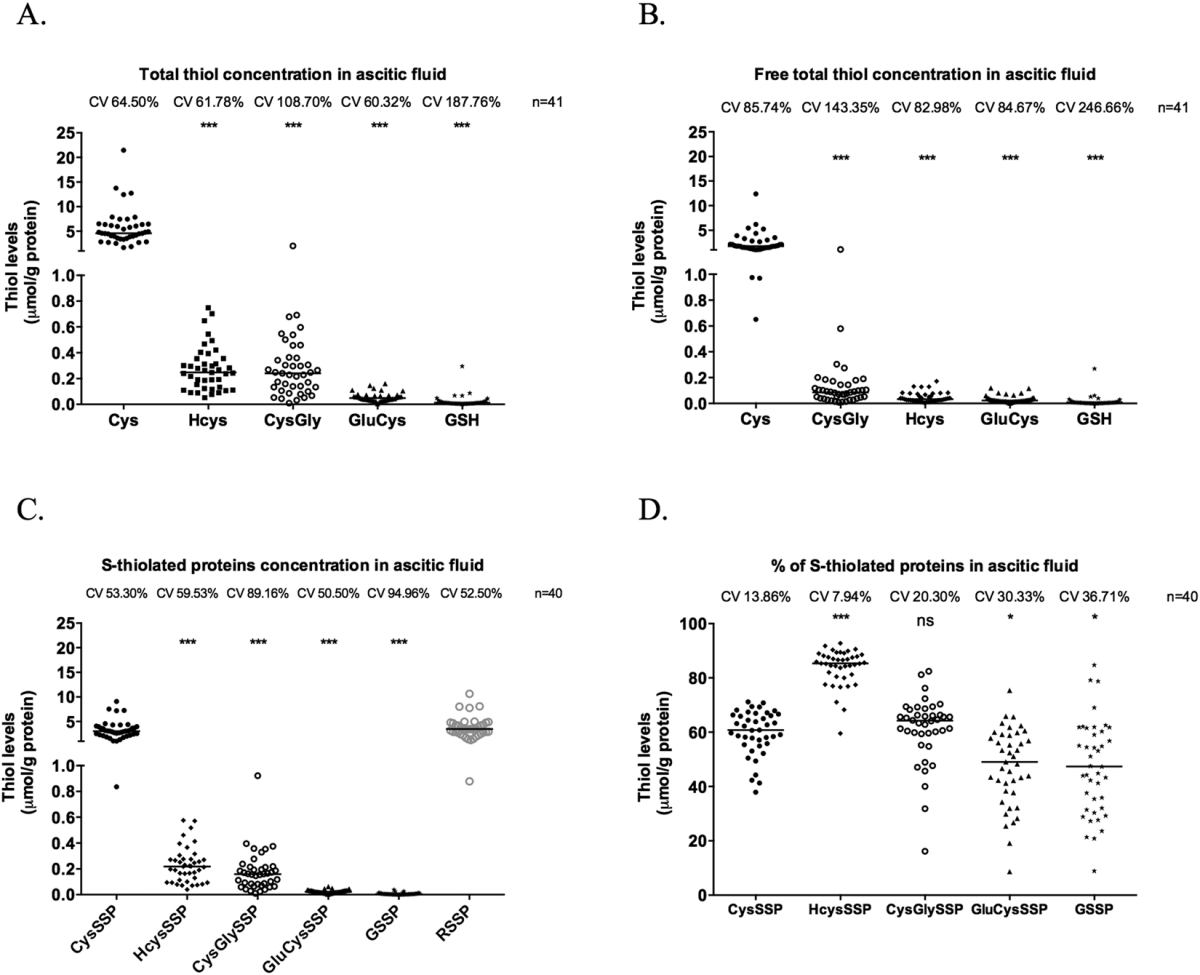
Cysteine is the prevalent thiol in ascitic fluid from patients with ovarian cancer. Thiols quantification in ascitic fluid from patients with ovarian cancer. (**A**) Total thiols concentration. (**B**) Free total thiols concentration. (**C**) Thiols concentration bound to proteins. (**D**) Percentage of thiols concentration bound to proteins. Cys – cysteine, HCys- Homocysteine, CysGly – Cysteinylglycine, GluCys – Glutamylcystein, GSH – Glutathione, CysSSP *– S*-cysteinylated proteins, HcySSP – S-homocysteinylated proteins, CysGlySSP – S-cysteinylglycinylated proteins, GluCysSSPS – Glutamylcysteinylated proteins, GSSP – S-glutathionylated proteins, RSSP – total S-thiolated proteins. Thiol concentration was normalized by protein concentration. Results are shown as median. The *represent the statistical significance in relation to Cysteine concentration. *p < 0.05, **p < 0.01, ***p < 0.001 (independent samples Kruskal Wallis One-way ANOVA with multiple comparisons).

As the ascitic fluid is an important component of ovarian tumour cells microenvironment, we disclosed if cysteine and other thiols were present in this biofluid derived from patients with ovarian cancer. Cysteine was the most prevalent thiol in the three pools, total (Fig. 8A), free (Fig. 8B) and S-cysteinylated proteins (Fig. 8C). HCys was mainly present bound to proteins, sequentially followed by Cys and CysGly, and then GluCys and GSH (Fig. 8D).

As the prevalent thiol in the ascitic fluid, cysteine might promote an ideal tumour microenvironment for cancer cells, possibly acting both as a redox buffer and as a modifier of proteins, mediating the adaptation or abrogating the effect of platinum salts, such as carboplatin.

## Discussion

Although the outcome prognosis of different histological types of ovarian cancer had been a matter of debate, it was shown that patients with OCCC had a significantly worse prognosis than patients with OSC when matched for age, stage, and level of primary cytoreduction^31^. In 2011, Lee *et al*. have shown that OCCC patients had a poorer prognosis than those with other histological subtypes of carcinoma, especially in advanced stages^32^. Moreover, while OCCC show intrinsic resistance to conventional platinum-based chemotherapy, OSC exhibit at first sensitiveness to platinum-based chemotherapy, though the majority of cases develop chemoresistance during the course of treatments^33,34^. In the present work, several ovarian cancer cell lines were used and major differences were found on cysteine dependence on copping with hypoxia and carboplatin. Importantly, ES2 cells and OVCAR8 presented a stronger dependence on cysteine metabolism in handling with hypoxic stress and carboplatin than OVCAR3. Considering A2780 and A2780 cisR cells under hypoxia upon carboplatin exposure, cysteine was advantageous for A2780 cisR but not for A2780 parental cells, reinforcing that cysteine accounts for the selective process of platinum resistant ovarian cancer cells. We must underline that OVCAR3 cells did not die more under hypoxia than ES2 cells. Instead, our results show that these cells manage hypoxic stress by different mechanisms. In fact, we observed that ES2 cells express higher levels of hypoxia-inducible factor 1-α (HIF-1α than OVCAR3 cells under both normoxia and hypoxia (Suppl. Fig. 3). Nevertheless, the ability to use cysteine proves to be advantageous under carboplatin exposure and allows a faster adaptive response to hypoxia, indicating that cysteine has a role in ovarian cancer cells survival in adverse environments. This evidence, on the other hand, points that cysteine metabolism is strongly related to a worse prognosis of ovarian cancer. It is plausible that the worse prognosis associated to the OCCC histological type is related to cysteine metabolism and or cysteine-dependent redox signalling process. However, we must test other OCCC cell lines in order to clarify the hypothesis that OCCC and OSC tumours are *a priori* metabolically different.

Moreover, we observed that the expression of CD133, a cancer stem cell marker^35^, was also increased in ES2, OVCAR8 and A2780 parental cells but not in OVCAR3 cells, upon cyclic exposure to hypoxia. Moreover, in these cells, carboplatin induced CD133 expression. This fact indicates that the activation of a pool of cancer stem cells underlies the adaptive capacity of these cells to hypoxia and drug resistance, where cysteine acts as a facilitator. Interestingly, upon carboplatin exposure, the already resistant A2780 cisR cells did not induce CD133 expression in the majority of treatments, whereas A2780 parental cells did. Those results support that CD133 expression is involved in the process of adaptation to carboplatin but it is lost when cells become chemoresistant, thus suggesting a role for CD133 in the adaptation to adverse environments.

Lopes-Coelho *et al*. have shown that, under normoxia, ES2 cells are more resistant to carboplatin than OVCAR3 and that the inhibition of GSH production by buthionine sulphoximine (BSO), sensitizes ES2 cells to carboplatin^23^. Our results suggest that the protective effect against carboplatin under hypoxia is mediated by cysteine. However, it is still unclear if GSH is involved in this protection under hypoxia. Moreover, our team has described recently that in normoxia and in glucose free media ES2 has higher levels of thiols than OVCAR3^23^. In the present study we cannot see that difference since we used different culture conditions, namely high glucose culture media. It is known that glucose can form adducts with glutathione, which can in part explain the observed differences^36^. Despite we have detected lower or equal levels of GSH in ES2 than in OVCAR3 cells, we also detected higher degradation of GSH in ES2, pointing a faster thiols’ turnover in this cell line indicated by the levels of cysteinylglycine (Cys-Gly)^37^.

In a general way, our results pointed to a tendency to decreased cell death levels with increased cell density. Recently, it was shown that cytosolic lipid droplets, which are known to protect cancer cells from starvation-induced death, increased in content with increased cell density in HeLa cells^38^. This observation could, in part, explain the protective effect of high initial cell densities observed in our cell models. However, in the initial cell density 5 × 10^3^ cells, we observed lower cell death levels than 2.5 × 10^4^. This could be due to cells dormancy that could ensure cells survival in a quiescent metabolic state, due to stressful conditions^39^ that could be imposed by such a decreased cell density. Moreover, the effect of cell density in the response to carboplatin is an issue that must be addressed in future studies using 3D models. One may expect that increased cell density will have an impact on cells capacity to adapt to carboplatin, since a larger cell population could present more heterogeneity, thus more adaptive potential. In fact, our preliminary results of spheroids models point out a protective effect of cysteine in ES2 but not in OVCAR3 upon carboplatin exposure (Suppl. Fig. 4). Interestingly, Lavi and colleagues^40^, through a mathematical model, showed how cancer cell density and cells alteration rate impact the heterogeneity over time, and its consequences on multidrug resistance. Moreover, in the context of ovarian cancer, Greene *et al*.^41^ have shown that local cell density and initial global cell density, are able to lead to significant differences in spatial growth, proliferation, and paclitaxel-induced apoptosis rates in OVCAR8 cells, thus having a role not only in cancer cells growth but also in drug response.

In the present work, a lower loss of Δψm under hypoxia plus cysteine compared to cysteine absence was expected. Although, it was not observed. Mitochondrial membrane potential (Δψm) has been considered a good indicator of cells health^30^, as it is critical for maintaining the physiological function of the respiratory chain to generate ATP in mitochondria. A significant loss of ΔΨm will deplete cells of energy with subsequent death. The obtained results are apparently contradictory to the data obtained with Annexin V/PI staining for cell death, in which, under hypoxia, cysteine was able to protect cells from death. However, annexin V labels apoptotic cells independently of the apoptotic pathways they underwent and as extrinsic apoptosis does not involve mitochondrial disruption it can be a plausible explanation for these results. On the other hand, to assess Δψm only adherent cells were used. Hence, cells that were dying faster under hypoxia without cysteine were not used to assess Δψm. Additionally, Tang *et al*. found that cancer cells were able to survive even after the cells had undergone critical apoptotic events such as mitochondrial fragmentation and dysfunction, nuclear condensation, cell shrinkage and activation of caspases^42^. They observed this apoptosis reversibility in various cancer cell lines and after different apoptotic stimuli^42^. Therefore, another interesting possibility is that, under hypoxia, cysteine is able to reverse apoptosis in ES2 cells.

With the exception of GSH, our results shown an overall increase of thiols concentration in serum from patients with ovarian neoplasms, regardless malignancy, compared to healthy donors. Strikingly, total and free levels of HCys distinguished all the three groups of individuals and free levels of Cys showed to be effective in distinguishing malignant from benign neoplasms. In disease, the literature has already shown that increased levels of HCys, Cys and CysGly in peripheral blood serum were already associated with ischemic heart disease^43^ and increased levels of CysGly^44^ and Cys^45^ were associated with increased risk of breast cancer^44^. Moreover, higher HCys levels were associated with greater risk of breast cancer only when combined with higher levels of Cys and with low levels of folate^45^. Differently, high plasma HCys levels were also associated with increased risk of colorectal cancer, whereas high Cys levels were associated with decreased risk for this disease^46^. Chiang and colleagues have confirmed the association between increased HCys levels and the risk of colorectal cancer adding that this increased risk was independent of oxidative stress indicators and antioxidant capacities^47^. In contrast, higher serum concentrations of Cys were associated with a significantly reduced risk of oesophageal squamous cell carcinomas and gastric cardia adenocarcinomas^48^. The assessment of different thiol pools total, free and protein linked confers an innovative approach in our work. We observed that the contribution of protein-S-thiolation was augmented in patients with ovarian neoplasms compared to healthy individuals. The reversible thiolation of proteins was already associated with the regulation of several metabolic processes such as enzyme activity, transport activity, signal transduction and gene expression^49^. Several proteins - such as Ras, Jun N-terminal kinase (JNK)- 2, AP-1, NF-kappaB, PKC, caspase, thioredoxin and p53^49,50^ - which are known to have important roles in cancer, are regulated by thiol oxidation^50^. Visscher *et al*. reported that many oncogenic mutations consist in an insertion of a novel cysteine in the protein sequence, as it happens in 12% of KRAS and 88% of FGFR mutations^51^. These facts suggest that newly introduced cysteines should play a role in tumourigenesis by contributing for tumour suppressor genes inactivation and oncogenes activation. Moreover, thiols can also interfere with epigenetic modulation of genes expression, which is pivotal in carcinogenesis^52^.

Importantly, our results have shown that cysteine levels are putative biomarkers for ovarian cancer early diagnosis. As seen, the total levels in peripheral blood of protein-S-cysteinylation distinguished healthy donors from patients with ovarian neoplasms (benign and malignant) and free cysteine distinguishes patients with ovarian benign tumours from patients with malignant tumours. This is undoubtedly a step forward in ovarian cancer research, as the late diagnosis is one of the most important barriers accounting for the poor outcome and high mortality.

We also observed that cysteine was the prevalent thiol in ascitic fluid from patients with ovarian cancer and that *S*- cysteinylation was the most abundant form of S-thiolated proteins showing, again, that cysteine is a relevant organic compound in ovarian cancer cells microenvironment.

We must emphasize that most samples analysed from patients with ovarian cancer were from OSC histotype. It would be interesting to assess thiols levels also in patients with OCCC. Albeit the limitation of the *in vitro* studies with a single OCCC cell line, we believe that the levels of cysteine and other thiols would be even higher in patients with this histological type compared to OSC, allowing faster disease progression and recurrence. Despite OCCC is frequently diagnosed at an initial stage^6^, in an extraovarian advanced stage the disease has poor prognosis given intrinsic chemoresistance to conventional platinum drugs^6^. However, what we undoubtably observe is that ovarian cancer cells that are better adapted to cysteine are more resistant to carboplatin effects mainly in hypoxia. The ability to take advantage of cysteine accounting for chemoresistance can be intrinsic, as OCCC, or acquired upon cyclic exposure to chemotherapy as happens with recurrent OSC.

**Figure 9:**
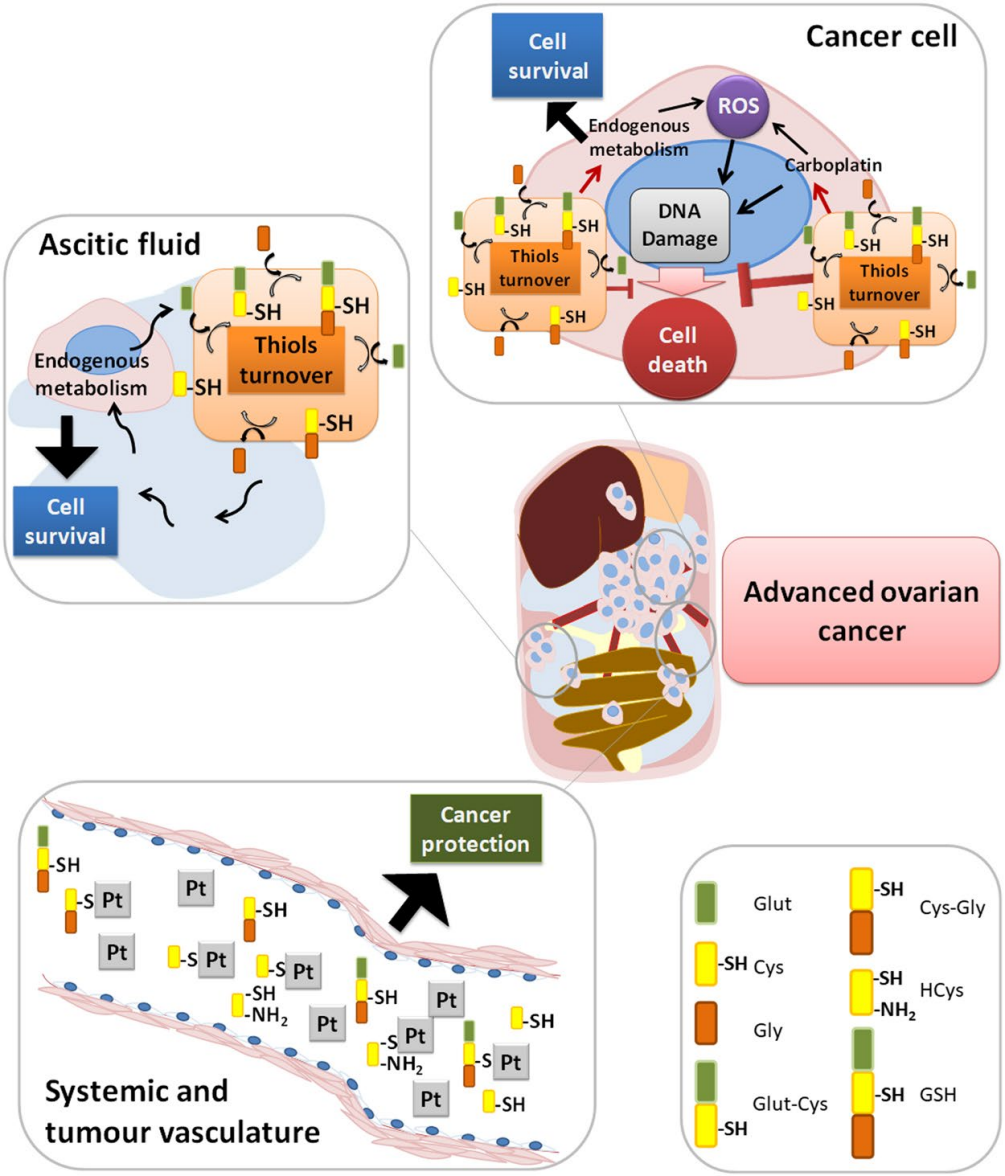
Role of cysteine in cells response to carboplatin and hypoxia adaptation. In cancer cells, cysteine allows adaptation to an adverse hypoxic microenvironment acting as a redox buffer, and to platinum-based chemotherapy as sulphur rapidly binds to carboplatin, avoiding DNA damage and evading apoptosis. Because of the presence of high cysteine concentrations in ascitic fluid from patients with ovarian cancer and also in serum from patients with ovarian malignant neoplasms, we propose that cysteine acts as a first protection barrier for malignant cells against hypoxia, having a role as a redox buffer. Results suggest a role of cysteine in ovarian malignant tumours and in disease progression, as cysteine is the prevalent thiol in ascitic fluid, an important compartment of tumour cells microenvironment.

Taken together, results suggest that cysteine protects cells from the adverse hypoxic microenvironment and from platinum-based chemotherapy, thus having a role in cancer progression. The high cysteine concentrations in ascitic fluid and in serum from patients with ovarian malignant tumours leads us to propose that cysteine acts as a first protection barrier for neoplastic cells against hypoxia, having a role as a redox buffer, and against chemotherapy, as sulphur rapidly binds to platinum-based cancer chemotherapy, thus preventing drug efficacy (Fig. 9). In metastatic peritoneal disease, cysteine rich ascites contributes for the perpetual maintenance of resistance to therapy, as cancer cells proliferate directly embedded in ascitic fluid (Fig. 9).

This paper highlights the role of cysteine underlying the resistance of ovarian cancer cells to hypoxia and carboplatin, which hinders cancer fighting. We are currently developing more studies to disclose if cysteine metabolic pathways can be elected as therapeutic targets to reverse resistance to carboplatin. Nevertheless, the best achievement of our study was the assessment of total free-cysteine and total protein-S-cysteinylation levels as promising strategy for ovarian cancer screening and early diagnosis. Moreover, cysteine levels in peripheral blood serum from ovarian cancer patients can be used to stratify patients according to risk therapy resistance allowing a more accurate prediction of disease outcome.

## Material and Methods

### Cell culture

Cell lines ES2 (CRL-1978), OVCAR-3 (HTB-161) and OVCAR-8 (CVCL-1629) were obtained from American Type Culture Collection (ATCC). Cell line A2780 sensitive (93112519) and A2780 cisplatin resistant (93112517) were obtained from Sigma Aldrich. Cells were maintained at 37 °C in a humidified 5% CO_2_ atmosphere. ES2, OVCAR3 and OVCAR8 cell lines were cultured in DMEM (41965–039, Gibco, Life Technologies) supplemented with 1% FBS (S 0615, Merck), 1% antibiotic-antimycotic (AA) (P06–07300, PAN Biotech). A2780 parental and A2780 cisR cells were cultured in RPMI 1640 (BE12-167F, Lonza) supplemented with 0.58 g/L of L-glutamine, 1% FBS (S 0615, Merck) and 1% antibiotic-antimycotic (AA) (P06-07300, PAN Biotech).

Cells were exposed to 0.4 mM L-Cysteine (102839, Merck) and/or exposed to hypoxia-induced conditions with 0.1 mM cobalt chloride (C8661, Sigma-Aldrich). Cobalt is a hypoxia mimicking agent used in *in vivo*^53^ and *in vitro*^54–56^ studies. Chemically, CoCl_2_ reacts with oxygen impairing its dissolution and oxygenation of aqueous solutions^57^ and is a way of impairing the availability of oxygen in culture media.

Prior to any experiment, cells were synchronized under starvation (culture medium without FBS) for 8 h at 37 °C and 5% CO_2_.

### Peripheral blood serum and ascitic fluid

After collection, the blood was allowed to clot at room temperature, during 15–30 min. The clot was removed by centrifuging at 3000 rpm for 5 min at 4 °C. Serum is the resulting supernatant, which were preserved at −80 °C.

After collection of the ascitic fluid, it was centrifuged at 1200 rpm for 2 min at room temperature, to remove the cells avoiding cell lysis. The supernatant was preserved at −80 °C.

The serum and ascitic fluid samples were collected from different individuals, with the exception of four serum and ascitic fluid samples, in which the patients were the same. The levels of thiols were determined in these biological samples, as described below.

### Cell death analysis

Cells (5 × 10^5^ cells/well) were seeded in 6-well plates and cultured in control condition and exposed to 0.4 mM L-cysteine and/or hypoxia induced with 0.1 mM cobalt chloride. Cells were collected at 16 h and 24 h after stimulation.

To test whether the protective effect of cysteine in ES2 and OVCAR3 cells is concentration-dependent, cells (5 × 10^5^ cells/well) were seeded in 6-well plates and cultured in control condition and exposed to 0.1 mM, 0.2 mM, 0.4 mM, 0.8 mM and 1.0 mM L-cysteine and/or hypoxia induced with 0.1 mM cobalt chloride. Cells were collected at 16 h after stimulation.

To test whether the protective effect of cysteine under hypoxia was dependent on initial cell density, 5 × 10^3^, 2.5 × 10^4^, 5 × 10^4^ and 1 × 10^5^ cells/cm^2^ were seeded in 24-well plates and cultured in control condition and exposed to 0.4 mM L-cysteine and/or hypoxia induced with 0.1 mM cobalt chloride. Cells were collected at 16 h after stimulation.

Cells were harvested and centrifuged at 1200 rpm for 3 min, followed by incubation with 1 μL annexin V-Alexa Fluor 488 (640906, BioLegend) in 100 μL annexin V binding buffer 1× (10 mM HEPES (pH 7.4), 0.14 M sodium chloride (NaCl), 2.5 mM calcium chloride (CaCl_2_) and incubated at room temperature in the dark for 15 min. After incubation, samples were rinsed with 0.1% (w/v) BSA (A9647, Sigma) in PBS 1× and centrifuged at 1200 rpm for 3 min. Cells were suspended in 200 μL of annexin V binding buffer 1× and 5 μL propidium iodide (50 μg/mL). Acquisition was performed with a FACScalibur (Becton Dickinson). Data were analysed with FlowJo software.

### Mitochondrial membrane potential (MMP)

The MMP was measured in ES2 and OVCAR3 cell lines using JC-1 (M34152, Molecular Probes, Life Technologies)^58^, a specific mitochondrial probe, which becomes red when it enters polarized mitochondria and it is green in cytoplasm when mitochondrial potential is lost avoiding its entrance.

Cells (5 × 10^5^ cells/well) were seeded in 6-well plates and cultured in control condition and exposed to 0.4 mM L-cysteine and/or 0.1 mM cobalt chloride and were collected at 16 h after stimulation. Live cells were then washed with phosphate-buffered saline 1×, incubated for 30 min with 5 μM JC-1 (37 °C, 5% CO_2_), washed with PBS 1×, trypsinized, and recovered in a final volume of 300 μL of PBS. A positive depolarized control was used, in which cells were previously incubated with 50 μM CCCP for 5 min (37 °C, 5% CO_2_) and then incubated with JC-1_._ Acquisition was performed in a FACScalibur (Becton Dickinson). Data were analysed with FlowJo software.

### Cells response to carboplatin

Cells (1 × 10^5^ cells/well) were seeded in 24-well plates and cultured in control condition and exposed to 0.4 mM L-cysteine and/or 0.1 mM cobalt chloride. Cells were also exposed to the previous conditions combined with carboplatin 25 μg/mL. Cells were collected after one cycle of stimulation (16 h per cycle) and two cycles of stimulation (which was preceded by a period without drugs of proximately 32 h between cycles). Cell death analysis was performed.

### CD133 quantification

Cells (2 × 10^5^ cells/well) were seeded in 12-well plates (ES2, OVCAR3 and OVCAR8) or 24-well plates (1 × 10^5^ cells/well) (A2780 and A2780 cisR) and cultured in control condition and exposed to 0.4 mM L-cysteine and/or 0.1 mM cobalt chloride. Cells were also exposed to the previous conditions combined with carboplatin 25 μg/mL. Cells were collected after one cycle of stimulation (16 h per cycle) and two cycles of stimulation (which was preceded by a period without drugs of proximately 32 h between cycles). Cells were collected with PBS 1x -EDTA 2 mM, centrifuged at 1200 rpm for 3 min, re-suspended and incubated with 1 μL of CD133 (AC133-PE human, Miltenyi Biotec) and 99 μL PBS-BSA 0.1% for 20 min in the dark 4 °C. Acquisition was performed in a FACScalibur (Becton Dickinson). Data were analysed with FlowJo software.

### Thiols quantification

Because thiols can impact protein function by the formation of intra- and inter-molecular disulfide bond(s) with proteins^59^, or may exist as free thiols, we measured both total and free thiol levels.

Thiols quantification was performed by high-performance liquid chromatography (HPLC). Cells (4 × 10^6^) were cultured in 75 cm^2^ tissue culture flasks in control conditions and exposed either to 0.4 mM L-cysteine and/or 0.1 mM cobalt chloride for 16 h. The supernatants and the lysates were stored at −80 °C. The assessment of the levels of cysteine (Cys), homocysteine (HCys), glutamylcysteine (GluCys), glutathione (GSH) and cysteinylglycine (CysGly) was performed according to Grilo and co-authors^60^ and adapted to cell culture. The detector was set at excitation and emission wavelengths of 385 and 515 nm, respectively. The mobile phase consisted of 100 mM acetate buffer (pH 4.5) and methanol [98:2 (v/v)]. The analytes were separated in an isocratic elution mode for 20 min, at a flow rate of 0.6 mL/min. The thiolomic profile was also quantified in peripheral blood serum from healthy individuals and patients with malignant and benign ovarian tumours and in ascitic fluid from patients with ovarian cancer, according to Grilo *et al*.^60^.

Regarding serum samples, 12 samples of healthy donors, 25 samples of patients with benign ovarian tumours and 30 samples of patients with malignant ovarian tumours were analysed.

Regarding ascitic fluid samples, 41 samples were analysed, with patients diagnosed with the following ovarian cancer histotypes: serous, (n = 32), mucinous (n = 3), endometriod (n = 1,) and without histotype information (n = 5).

### Statistical analysis

Data are presented as the mean ± SD and all the graphics were done using the PRISM software package (PRISM 6.0 for Mac OS X; GraphPad software, USA, 2013). Assays were performed with, at least, 3 replicates per experimental condition. For comparisons of two groups, two-tailed independent-samples T-test was used. For comparison of more than two groups, One-way analysis of variance (ANOVA) with Tukey’s multiple-comparisons post hoc test was used. To assess the existence of a linear relationship between two variables, two-tailed Pearson correlation was used. To assess differences in cysteine and GSH levels in peripheral blood and in ascitic fluid, 2-sided independent samples Kruskal-Wallis 1-way ANOVA with pairwise comparisons was performed, where the adjusted significance was considered. Statistical significance was established as *p* < 0.05. All statistical analyses were performed using the IBM Corp. Released 2013. IBM SPSS Statistics for Macintosh, Version 22.0. Armonk, NY: IBM Corp. software.

### Study approval

All the experiments were developed according to relevant guidelines and regulations.

The human samples were collected under informed consent of patients and blood donors from Instituto Português de Oncologia de Lisboa, Francisco Gentil, EPE. The study protocol was approved by the Ethical Committee IPOLFG (references: UIC/1080 and UIC/1082).

### Data availability

All data generated and analysed during this study are included in the article and in supplementary information. Cell lines and reagents are commercially available.

## Electronic supplementary material

Supplementary information
