# Supplementary material for: Cysteine allows ovarian cancer cells to adapt to hypoxia and to escape from carboplatin cytotoxicity
Source: Sci Rep. 2018 Jun 22;8:9513. doi: 10.1038/s41598-018-27753-y (PMC6015047; doi:10.1038/s41598-018-27753-y)
Supplement: Supplementary file 1 — Supplementary information [file 41598_2018_27753_MOESM1_ESM.pdf]

**Cysteine allows ovarian cancer cells to adapt to hypoxia and to escape from carboplatin cytotoxicity**

Sofia C. Nunes[1,2], Cristiano Ramos[1,2], Filipa Lopes-Coelho[1,2], Catarina O. Sequeira[1], Fernanda Silva[1,2], Sofia Gouveia-Fernandes[1,2], Armanda Rodrigues[1,2], António Guimarães[3], Margarida Silveira[4], Sofia Abreu[5,6], Vítor E. Santo[5,6], Catarina Brito[5,6], Ana Félix[1,7], Sofia A. Pereira[1], Jacinta Serpa[1,2*]

1. CEDOC, Chronic Diseases Research Centre, NOVA Medical School|Faculdade de Ciências Médicas, Universidade NOVA de Lisboa, Campo dos Mártires da Pátria, 130, 1169-056 Lisboa, Portugal

2. Unidade de Investigação em Patobiologia Molecular do Instituto Português de Oncologia de Lisboa Francisco Gentil (IPOLFG), Rua Prof Lima Basto 1099-023, Lisboa, Portugal

3. Serviço de Oncologia Médica do Instituto Português de Oncologia de Lisboa Francisco Gentil (IPOLFG), Rua Prof Lima Basto 1099-023, Lisbon, Portugal

4. Serviço de Patologia Clinica do Instituto Português de Oncologia de Lisboa Francisco Gentil (IPOLFG), Rua Prof Lima Basto 1099-023, Lisbon, Portugal

5. Instituto de Biologia Experimental e Tecnológica, Apartado 12, 2780-901 Oeiras, Portugal

6. Instituto de Tecnologia Química e Biológica António Xavier, Universidade Nova de Lisboa, Av. da República, 2780-157 Oeiras, Portugal

7. Serviço de Anatomia Patológica do Instituto Português de Oncologia de Lisboa Francisco Gentil (IPOLFG), Rua Prof Lima Basto 1099-023, Lisbon, Portugal

Sofia C. Nunes; E-mail: sofia.nunes@nms.unl.pt

Cristiano Ramos; E-mail: ramos.cristiano.93@gmail.com

Filipa Lopes-Coelho; E-mail: filipa.coelho@nms.unl.pt

Catarina O. Sequeira; E-mail: catarina.sequeira@nms.unl.pt

Fernanda Silva; E-mail: fernanda.silva@nms.unl.pt

Sofia Gouveia-Fernandes; E-mail: sofia.gopf@live.com.pt

Armanda Rodrigues; E-mail: maria.rodrigues@nms.unl.pt

António Guimarães; E-mail: aguimaraes@ipolisboa.min-saude.pt

Margarida Silveira; E-mail: msilveira@ipolisboa.min-saude.pt

Sofia Abreu; E-mail: sabreu@ibet.pt

Vitor E. Santo; E-mail: vitorespsanto@gmail.com

Catarina Brito; E-mail: anabrito@ibet.pt

Ana Félix; E-mail: ana.felix@nms.unl.pt

Sofia A. Pereira; E-mail: sofia.pereira@nms.unl.pt

*Jacinta Serpa; E-mail: jacinta.serpa@nms.unl.pt

A.

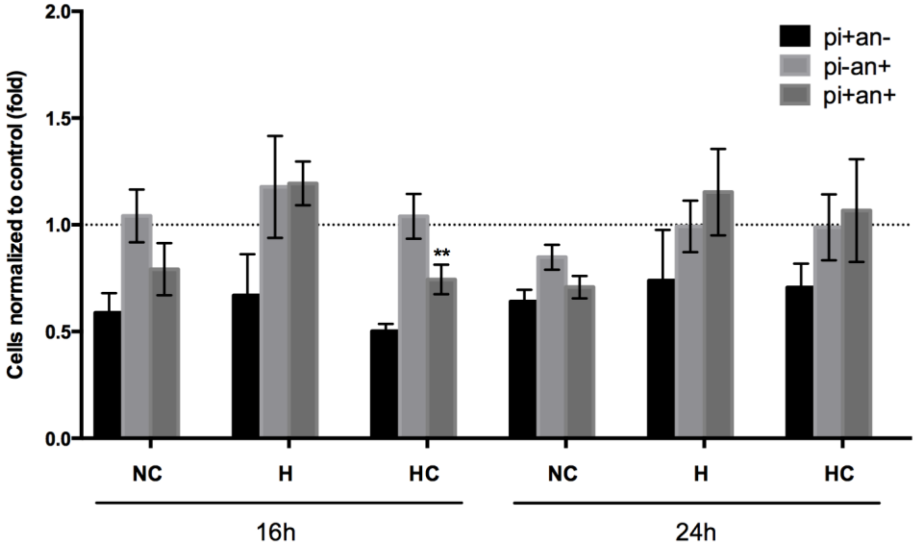


B.

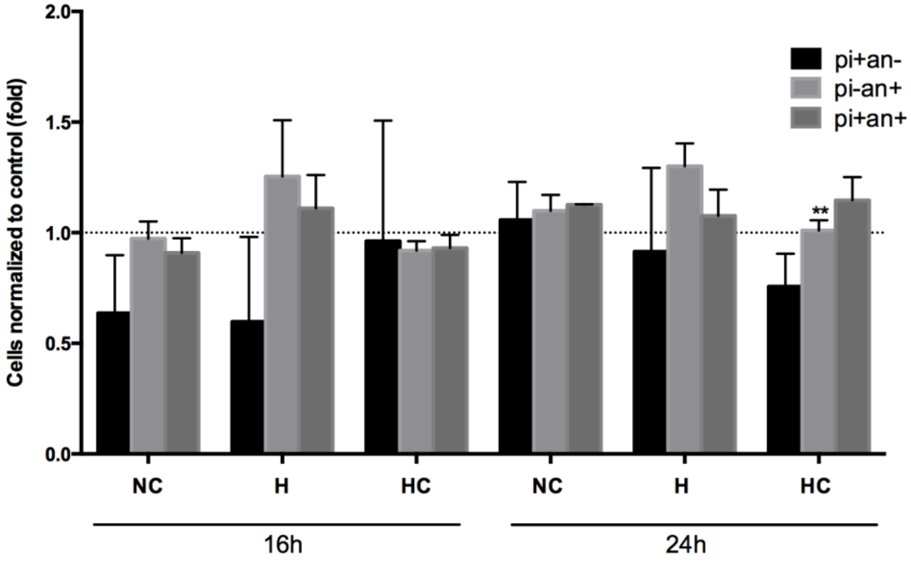


**Supplementary figure 1. Effect of cysteine and hypoxia in ES2 and OVCAR3 cells death.**

Percentage of pi positive cells (necrosis), Annexin V positive cells (early apoptosis) and pi/annexin V positive cells in A. ES2 cells and B. OVCAR3 cells showing that OVCAR3 cells present significant differences in early apoptosis among hypoxia with and without cysteine supplementation ($p$=0.009) after 24 h of assay.

N - Normoxia; NC - Normoxia supplemented with cysteine; H - Hypoxia; HC - Hypoxia supplemented with cysteine. Results are shown as mean ± SD. The asterisks represent the statistical significance among hypoxia with and without cysteine supplementation. *p<0.05, **p<0.01, ***p<0.001 (One-way ANOVA with post hoc Tukey tests).

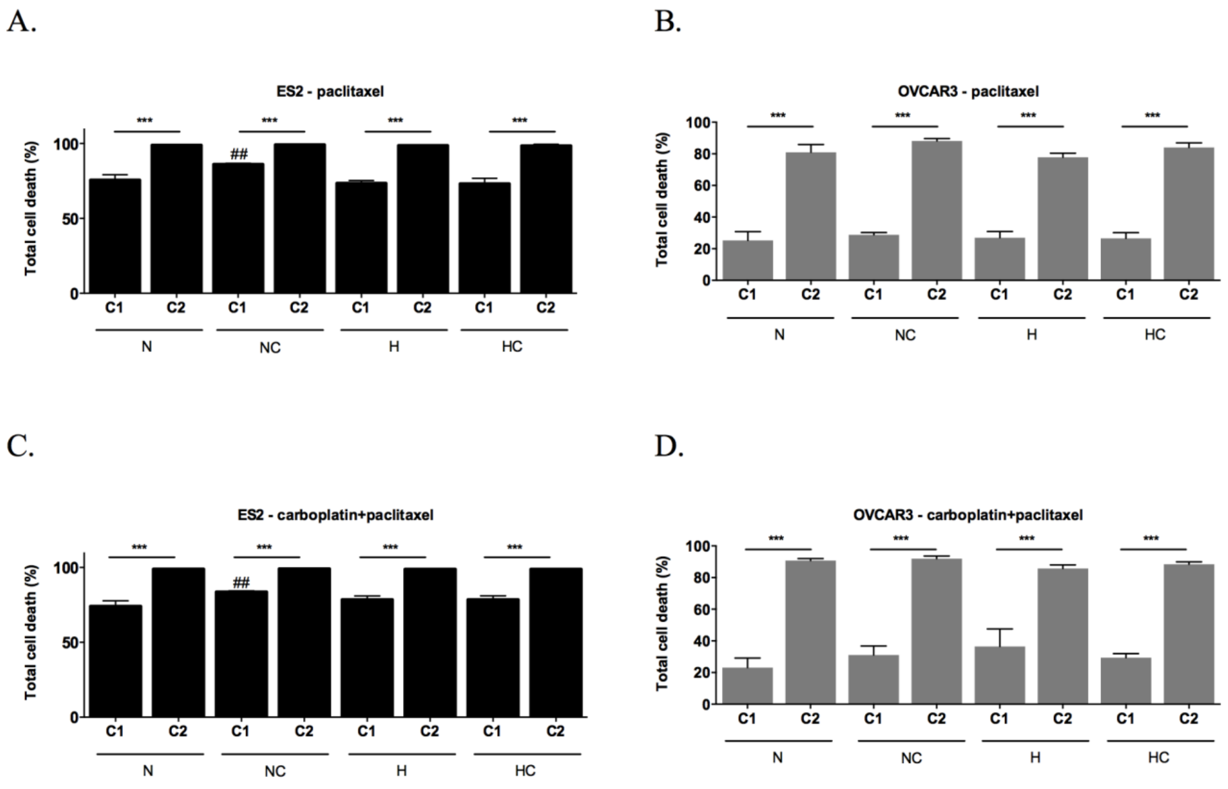


**Supplementary figure 2. Effect of cysteine and hypoxia in ES2 and OVCAR3 cells response to paclitaxel alone or in combination with carboplatin.**

Percentage of cell death levels in the presence of paclitaxel (10μg/mL) for A. ES2 cells and B. OVCAR3 cells, and in the presence of paclitaxel in combination with carboplatin for C. ES2 cells and D. OVCAR3 cells.

N – Normoxia; NC – Normoxia supplemented with cysteine; H – Hypoxia; HC – Hypoxia supplemented with cysteine. Results are shown as mean ± SD. The asteriscs represent statistical significance among cycles of treatments and cardinals represent statistical significance compared to the control (normoxia) within the same cycle of treatments. In the first cycle of treatments, with paclitaxel,

ES2 cells presented higher levels of cell death under normoxia with cysteine supplementation compared to the other treatments (NC vs N $p$=0.007, NC vs H $p$=0.002 and NC vs HC $p$=0.002, Tukey tests). However, in the second cycle, there were no differences among treatments ($p$>0.05, One-way ANOVA) and cells almost reached 100% of cell death (figure supplement 1A). For OVCAR3 cells, there were differences only in the second cycle of treatments, in which cysteine was disadvantageous for cells under normoxia compared to hypoxia without cysteine supplementation ($p$=0.018, Tukey test) (figure supplement 1B). With Paclitaxel in combination with carboplatin, cysteine was disadvantageous for ES2 cells under normoxia (N vs NC p=0.008, Tukey test) in the first cycle of treatments, but no differences were observed among treatments with two cycles of treatments (figure supplement 1C). With both drugs, for OVCAR3, there were differences only in the second cycle of treatments, in which normoxia with ($p$=0.0010, Tukey test) or without cysteine supplementation ($p$=0.031, Tukey test) were disadvantageous compared to hypoxia without cysteine supplementation (figure supplement 1D). For both cells lines, cell death levels increased from the first to second cycle of treatments in all treatments ($p$=0.000, Tukey tests).*p<0.05, **p<0.01, ***p<0.001 (One-way ANOVA with post hoc Tukey tests).

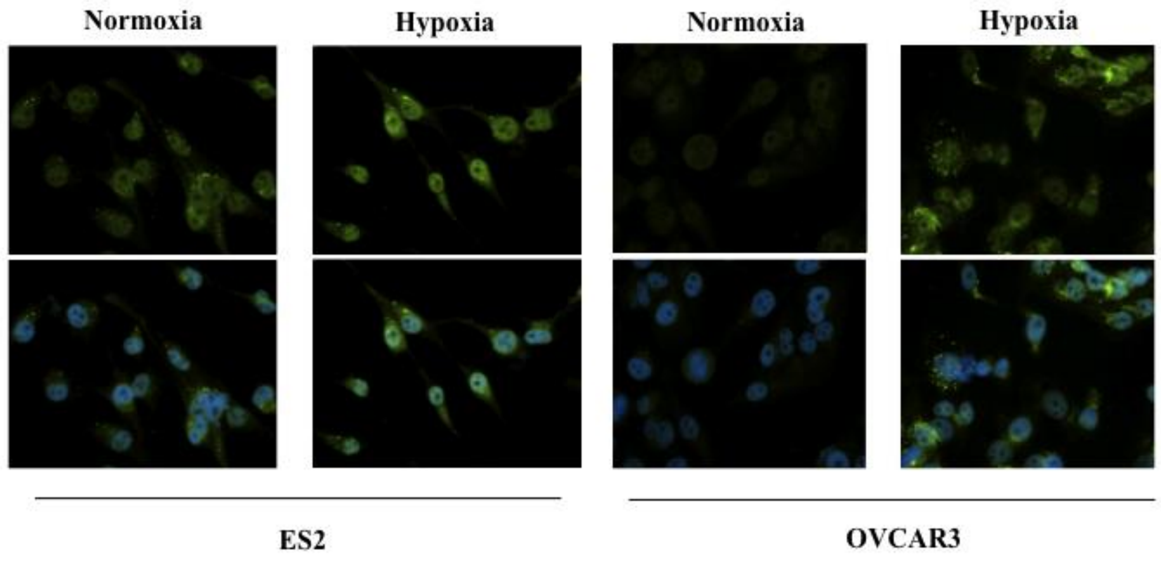


**Supplementary figure 3. ES2 cells present higher levels of Hif-1α expression than OVCAR3 cells.**

Immunofluorescence analysis of HIF-1α expression (green) under normoxia and hypoxia for ES2 and OVCAR3 cells. Nuclei were stained with DAPI (blue).

Cells (5× $10^4$ cells/well) were seeded in 24-well plates and cultured either in control condition or exposed to 0.100mM cobalt chloride. Cells were collected after 16 hours of conditions and Anti-Hif-1α (anti-goat antibody from SICGENE AB0112-200) was used. Cells were fixed with 2% paraformaldehyde for 15 min at 4 °C and permeabilized with PBS-BSA 0.1%-0.01% Triton for 30 min. Cells were incubated with primary antibody overnight at 4ºC (diluted in PBA-BSA 0.1%, 1:100). Samples were incubated with secondary antibody for 2 h at room temperature. Secondary antibody anti-goat Alexa Fluor® 488 (1:1000 in PBA-BSA 0.1%. Antibody from Jackson ImmunoResearch Laboratories) was used. The slides were mounted in VECTASHIELD media with DAPI (4′-6-diamidino-2-phenylindole) (Vector Labs) and examined by standard fluorescence microscopy using a Nikon Instruments Eclipse Ti-S Inverted Microscope (Hamamatsu digital camera C10600 ORCA-$R^2$). Images were acquired and processed with NIS-Elements AR-3.2 (magnification 400x).

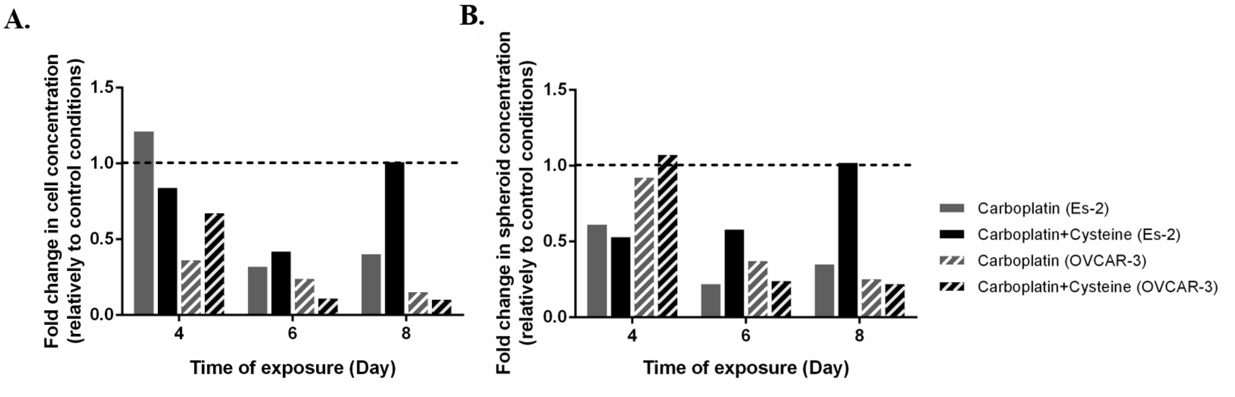


**Supplementary figure 4. ES2 (OCCC) and OVCAR3 (OSC) spheroid exposure to chemotherapeutic treatment: protective effect of cysteine.**

Single cell suspension of OVCAR3 and ES2 cells (5x$10^6$ cell/mL) were inoculated in spinner vessels and maintained under 60-80 rpm agitation to induce aggregation, according to Santo et al. (J Biotech, 2016). Spheroid cultures were challenged with 2 cycles of exposure with 25µg/mL carboplatin, with or without supplementation with 0.4µM cysteine (on day 0 and 4), and compared to control cultures without chemotherapy treatment, with or without cysteine supplementation. Cell concentration (A), determined by detection of cellular DNA, and spheroid concentration (B), determined by phase contrast microscopy and automatic evaluation using ImageJ software (as described in Santo et al., J Biotech,

2016) were evaluated along drug exposure time. All data is presented as fold change relative to the respective control condition.
